# Increased Risk of Acute Pancreatitis in Patients with Rheumatoid Arthritis: A Population-Based Cohort Study

**DOI:** 10.1371/journal.pone.0135187

**Published:** 2015-08-11

**Authors:** Chi Ching Chang, Chi Sheng Chiou, Hsiu Li Lin, Li Hsuan Wang, Yu Sheng Chang, Hsiu-Chen Lin

**Affiliations:** 1 Department of Internal Medicine, School of Medicine, College of Medicine, Taipei Medical University, Taipei, Taiwan; 2 Division of Rheumatology, Immunology and Allergy, Department of Internal Medicine, Taipei Medical University Hospital, Taipei, Taiwan; 3 Department of Neurology, General Cathay Hospital, Sijhih Branch, New Taipei City, Taiwan; 4 Graduate Institute of Biomedical Informatics, College of Medical Science and Technology, Taipei Medical University, Taipei, Taiwan; 5 School of Pharmacy, College of Pharmacy, Taipei Medical University, Taipei, Taiwan; 6 Department of Pharmacy, Taipei Medical University Hospital, Taipei, Taiwan; 7 Division of Allergy, Immunology, and Rheumatology, Department of Internal Medicine, Shuang Ho Hospital, Taipei Medical University, New Taipei City, Taiwan; 8 Department of Pediatrics, School of Medicine, College of Medicine, Taipei Medical University, Taipei, Taiwan; 9 Department of Laboratory Medicine, Taipei Medical University Hospital, Taipei, Taiwan; University of Szeged, HUNGARY

## Abstract

The study was conducted to determine whether patients with rheumatoid arthritis (RA) are at increased risk of acute pancreatitis compared with those without RA and to determine if the risk of acute pancreatitis varied by anti-RA drug use. We used the large population-based dataset from the National Health Insurance (NHI) program in Taiwan to conduct a retrospective cohort study. Patients newly diagnosed with RA between 2000 and 2011 were referred to as the RA group. The comparator non-RA group was matched with propensity score, using age and sex, in the same time period. We presented the incidence density by 100,000 person-years. The propensity score and all variables were analyzed in fully adjusted Cox proportional hazard regression. The cumulative incidence of acute pancreatitis was assessed by Kaplan-Meier analysis, with significance based on the log-rank test. From claims data of one million enrollees randomly sampled from the Taiwan NHI database, 29,755 adults with RA were identified and 119,020 non- RA persons were matched as a comparison group. The RA cohort had higher incidence density of acute pancreatitis (185.7 versus 119.0 per 100,000 person-years) than the non-RA cohort. The adjusted hazard ratio (HR) was 1.62 (95% CI [confidence interval] 1.43–1.83) for patients with RA to develop acute pancreatitis. Oral corticosteroid use decreased the risk of acute pancreatitis (adjusted HR 0.83, 95% CI 0.73–0.94) but without a dose-dependent effect. Current use of disease modifying anti-rheumatic drugs or tumor necrosis factor blockers did not decrease the risk of acute pancreatitis. In conclusion, patients with RA are at an elevated risk of acute pancreatitis. Use of oral corticosteroids may reduce the risk of acute pancreatitis.

## Introduction

Acute pancreatitis is a common disorder and often associated with significant co-morbidities and substantial mortality rates. The fatality rate of the disease ranges approximately from 2% to 5% [1–3]. The etiology of acute pancreatitis is undoubtedly multifactorial. Epidemiological studies have shown that gallbladder stones (cholelithiasis), hypertriglyceridemia, obesity, viral hepatitis, and lifestyle are significant risk factors associated with acute pancreatitis. The proportion of cases of unknown etiology (idiopathic pancreatitis) is being progressively reduced as more genetic factors are discovered [4–7]. Clinical or biochemical autoimmune stigmata are present in 40% of patients with idiopathic pancreatitis [8]. Current evidence strongly suggests rheumatoid arthritis, Sjogren’s syndrome, and inflammatory bowel disease are frequently associated with autoimmune pancreatitis [9–12]. Whereas most cases present at a chronic stage, there are reported cases of acute pancreatitis, in particular after steroid withdrawal [13]. Drug toxicity can be an additional etiologic agent for acute pancreatitis. The long-term use of corticosteroids and immunosuppressives has been well associated with pancreatitis [14–17]. Additionally, limited studies have observed an association between anti-rheumatoid arthritis (RA) drugs and the risk of acute pancreatitis. Therefore, we intended to determine the risk of developing acute pancreatitis among RA patients with various anti-RA drugs.

RA is a chronic systemic inflammatory disease that occurs in 0.5% to 1.0% of the adult population worldwide [18–19]. RA is often characterized by swollen joints, pain, and decreased physical function. But less understood are the many manifestations of additional health conditions that are associated with RA and its treatments.

The prevalence rate of RA in Taiwan has increased steadily from 57.7/100,000 in 2000 to 99.6/100,000 in 2007 [20]. The chronic, debilitating, autoimmune nature of RA affects the patient directly or indirectly in nearly all organ systems, from cardiovascular problems and infections to depression and gastrointestinal ulcers [21]. Our knowledge of RA-related acute pancreatitis is mostly based on individual case reports or small case series [22–25]. Despite its rarity, acute pancreatitis can be a life-threatening complication of RA if not treated appropriately. The risk of acute pancreatitis in patients with RA has not been well established. Assessment of the risk of RA patients developing acute pancreatitis requires study of a large population in a cohort study. We used the large population-based dataset available from the National Health Insurance (NHI) program in Taiwan to conduct a retrospective cohort study to evaluate the risk of acute pancreatitis in RA patients. The aim of this study is to estimate the incidence of acute pancreatitis among patients with RA relative to a propensity score-matched population without RA. We also investigated the role of anti-RA drugs including steroid, disease modifying anti-rheumatic drugs (DMARDs), and Tumor Necrosis Factor (TNF)-blockers in the occurrence of acute pancreatitis in RA patients.

## Materials and Methods

### Data sources

This study was a retrospective cohort study. We obtained a dataset of reimbursement claims from the National Health Research Institutes (NHRI), which manages the National Health Insurance (NHI) databank. The dataset represented the registry of a randomly sampled cohort of one million people from the 23,753,407 people enrolled in the NHI system with claims from 2000 to 2011. There were 23,805,568 residents in Taiwan in 2000, and the NHI program covered the medical care for 99.8% of these residents. The claims databank is one of the largest and most comprehensive nationwide population-based datasets available in the world and includes diagnostic codes of individual diseases, pharmaceutical prescriptions, and medical expenditures for outpatient and inpatient care since 1996. The dataset was obtained from the Longitudinal Health Insurance Database (LHID) of the NHI program. The NHRI declared that there were no statistically significant differences in age, sex, and area distribution between the patients in the sampled group and the original dataset population. The ratio of men to women was 104.7:100. The percentages of individuals aged 0 to 14 years, 15 to 64 years, ≥65 years were 20.81%, 70.39%, and 8.8%, respectively. Most of the individuals (44.6%) with information available in the NHI database lived in northern Taiwan. The LHID is a nationwide population-based data set that provides outpatient and inpatient claims for 11 years of follow-up and is, therefore, an excellent resource for evaluating the risk of acute pancreatitis in patients with RA. Because the LHID dataset consists of de-identified and secondary data released to the public for research, the study was exempt from full review by the Institutional Review Board of Taipei Medical University.

### Criteria and definition

We identified patients aged 18 years or older with RA based on the ICD-9-CM (International Classification of Diseases 9th Revision, Clinical Modification) code 714.0, who were newly diagnosed between 2000 and 2011. In Taiwan, RA is generally diagnosed by specialists in rheumatology based on clinical symptoms, radiographic changes, and identification of serum rheumatoid factor. In this study, we selected patients who had received two or more RA diagnoses with at least one being made by a specialist in rheumatology. In addition, we only included RA cases if they had been prescribed at least one type of DMARD. For each RA case, we selected four individuals without medical claims for RA in order to increase the statistical power and who were then matched with a propensity score to avoid selection bias [26]. The propensity score match included age (at enrolled year) and sex, which was matched with the RA group. The outcome of interest that was identified was patients who were categorized as having developed acute pancreatitis (ICD-9-CM codes 577.0). To measure the incidence of acute pancreatitis, the RA group and the non-RA group were followed up until acute pancreatitis was identified by the end of 2011 or until censoring occurred because of death or withdrawal from the insurance program. Patients who had been diagnosed with acute pancreatitis or chronic pancreatitis before RA was diagnosed were excluded from this study.

### Comorbidities and medications

Risk factors for acute pancreatitis were considered as potential confounders and were adjusted during the analysis. These factors included hyperlipidemia, alcoholism, gallbladder stones, and viral hepatitis. The corresponding ICD-9-CM codes for these diseases are 272.0–272.4, for hyperlipidemia; 291, 303.0, 303.9, 305.0, 357.5, 425.5, 535.3, 571.0–571.3, 790.3, 980.0, V113, V791, for alcoholism; 574 for gallbladder stone; and 070, V026 for viral hepatitis. Medications for RA were classified as DMARD (cyclosporine, tacrolimus, mycophenolate mofetil, azathioprine, sulfasalazine, hydroxychloroquine, methotrexate, leflunomide) TNFs-blockers (ie, etanercept, adalimumab) or oral corticosteroids which were classified to daily prednisolone equivalent doses (≤ 5 mg, 5.01−10 mg, 10.01–15 mg and >15 mg) depending on the various kinds of corticosteroids prescribed.

### Statistical analysis

The SAS 9.1 statistical package (SAS Institute Inc., Cary, NC, USA) was used to perform all analyses in this study. Because these two groups were normally distributed, we examined differences in continuous variables between groups by student's t-test and used Pearson's χ^2^-square test to examine differences in dichotomous variables that were potential confounders between the two groups.

The incidence density is represented as per 100,000 person-years. Years were measured from the date of diagnosis of RA or date of enrollment in this study to the occurrence of acute pancreatitis. Then, the cumulative years of each case of acute pancreatitis were used to obtain the person-years. The propensity score for patients with and without RA was calculated using all of variables (comorbidities, sex, age, years from entry) in a fully adjusted Cox proportional hazard regression for acute pancreatitis. Crude and adjusted hazard ratios (HR) are presented along with their 95% confidence interval (CI). The cumulative incidence of acute pancreatitis was assessed by Kaplan-Meier analysis, with significance based on the log-rank test. The results of all statistical tests were considered significant if the two-sided *p*-value was ≤ 0.05.

## Results

### Baseline characteristics of the study population

**Fig 1:**
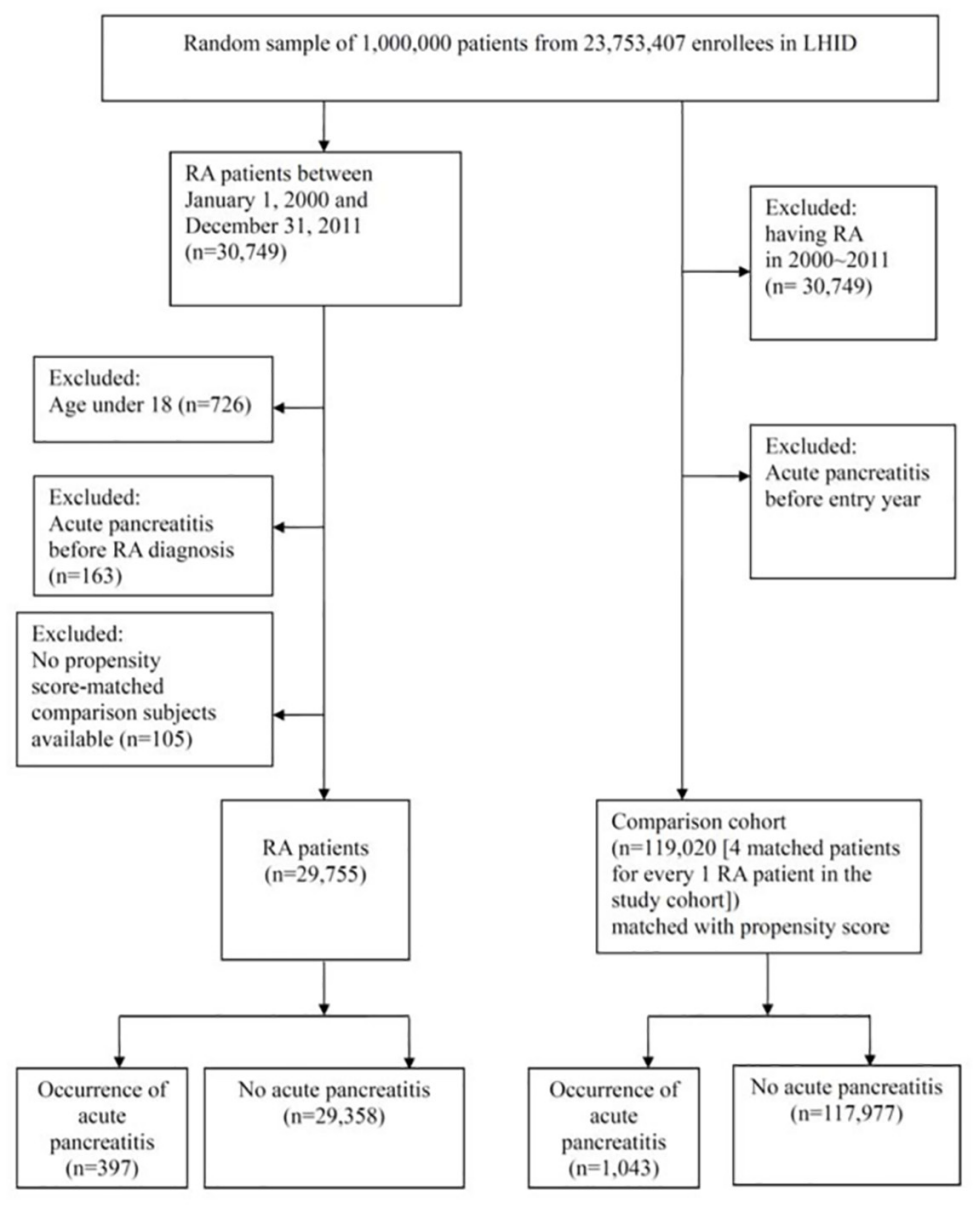
Procedures used for selection of cases. Footnotes: LHID*: Longitudinal Health Insurance Database. RA**: rheumatoid arthritis.

Initially, we identified 30,749 patients with RA from the LHID. The final number of enrolled patients was 29,755 after exclusion for the above mentioned criteria (Fig 1). Eligible comparison patients included 119,020 in the propensity score (PS)-matched non-RA group, with similar sex and age distributions (mean age, 53.15 years) (Table 1). There were no significant differences in the prevalence of hyperlipidemia, alcoholism, gallbladder stones, or viral hepatitis between the RA group and the PS-matched non-RA group. The overall incidence of acute pancreatitis in the RA group was higher than in the non-RA group (1.33% vs.0.88%).

**Table 1:** Demographic characteristics of RA patients and PS-matched comparison group.

Variables	Comparison group^a^	Patients with RA	P
	(N = 119,020)	(N = 29,755)	
Acute pancreatitis	1,043 (0.88%)	397 (1.33%)	<0.001
Age (mean±SD)	53.14 ± 15.79	53.15 ± 15.79	0.93
Sex (male/female)	41,658 / 77,362	10,424 / 19,331	0.92
Comorbidity			
Hyperlipidemia	46,876 (39.38%)	11,715 (39.37%)	0.96
Alcoholism	2,556 (2.15%)	652 (2.19%)	0.64
Gallbladder stone	43,302 (36.38%)	10,822 (36.37%)	0.97
Viral hepatitis	12,465 (10.47%)	3,146 (10.57%)	0.62

PS-matched: propensity score-matched; RA: rheumatoid arthritis.

^a^Comparison group: patients without RA and PS-matched.

### Acute pancreatitis based on multivariate Cox proportional hazard analysis

The crude hazard ratio (HR) of developing acute pancreatitis during the follow-up period for patients with RA was 1.57 (95% CI 1.39 to 1.77) compared with non-RA patients (Table 2). After adjusting for patients’ gender, age, and other comorbid medical condition, the hazard of developing acute pancreatitis was 1.62 (95% CI 1.43–1.83) times greater for patients with RA compared with non RA patients (Table 2), suggesting that RA is an independent factor in determining the future risk of acute pancreatitis. The RA cohort had higher incidence density of acute pancreatitis (185.7 versus 119 per 100,000 person-years) than the non-RA cohort. In addition, patients in both cohorts who were of older age or male gender also had an increased risk of developing acute pancreatitis.

**Table 2:** Incidence density and hazard ratios for occurrence of acute pancreatitis in the two different groups.

Variables	n	Cases	Person-years	Incidence density^a^	Crude HR (95%CI)	Adjusted HR (95%CI)
Rheumatoid arthritis						
No	119,020	1,043	876,258	119	1.00	1.00
Yes	29,755	397	213,756	185.7	1.57*** (1.39~1.77)	1.62*** (1.43~1.83)
Gender						
Female	96,693	780	704,812	110.7	1.00	1.00
Male	52,082	660	385,102	171.4	1.55*** (1.39~1.72)	1.17* (1.03~1.33)
Age (years)					P for trend<0.05	P for trend<0.05
18–30	11,792	41	88,287	46.4	1.00	1.00
31–50	53,781	385	391,251	98.4	2.12*** (1.53~2.92)	1.63*** (1.17~2.27)
51–65	47,175	444	336,044	132.1	2.88*** (2.09~3.97)	2.54*** (1.82~3.55)
> 65	36,027	570	274,332	207.8	4.51*** (3.29~6.19)	4.12*** (2.96~5.72)

CI: confidence interval; HR: hazard ratio. Adjusted for age, sex, alcoholism, viral hepatitis, hyperlipidemia, gallbladder stones, and 10 types of anti-RA drugs (DMARDs, TNF-blockers, corticosteroids).

^a^ Incidence density per 100,000 person-years

*p<0.05

***p<0.001

### Influence of anti-RA drugs on the risk of acute pancreatitis

Approximately 89% of patients had current exposure to glucocorticoid therapy. Exposure to current glucocorticoid therapy was associated with a decreased risk of acute pancreatitis (adjusted HR 0.83, 95% CI 0.73–0.94). DMARD therapy did not have a beneficial effect in reducing the risk of acute pancreatitis. Furthermore, treatment with TNF-blockers did not decrease risk of acute pancreatitis (Table 3).

**Table 3:** The prescription of various anti-RA drugs in the two groups and the risk of acute pancreatitis.

Variables	Comparison group^a^	Patients with RA	Crude HR (95%CI)	Adjusted HR (95%CI)
Cyclosporine	137 (0.12%)	356 (1.20%)	1.13 (0.51~2.53)	1.31 (0.53~3.19)
Tacrolimus	73 (0.06%)	13 (0.04%)	2.49 (0.62~9.99)	1.58 (0.23~11.06)
Mycophenolate mofetil	122 (0.06%)	30 (0.10%)	1.43 (0.36~5.71)	0.78 (0.11~5.46)
Azathioprine	302 (0.25%)	381 (1.28%)	1.19 (0.59~2.37)	1.20 (0.59~2.47)
Sulfasalazine	506 (0.43%)	3,109 (10.45%)	1.24 (0.91~1.68)	1.18 (0.81~1.71)
Hydroxychloroquine	913 (0.77%)	4,199 (14.11%)	1.16 (0.88~1.52)	1.01 (0.73~1.40)
Methotrexate	559 (0.47%)	2,077 (6.98%)	0.94 (0.63~1.41)	0.81 (0.49~1.33)
Leflunomide	23 (0.02%)	411 (1.38%)	0.65 (0.21~2.02)	0.63 (0.18~2.20)
TNF-blockers	0 (0%)	285 (0.96%)	0.64 (0.16~2.55)	0.66 (0.15~2.97)
Corticosteroids	91,026 (76.48%)	26,479 (88.99%)	0.92 (0.81~1.03)	0.83*** (0.73~0.94)

RA: rheumatoid arthritis. Adjusted for age, gender, alcoholism, viral hepatitis, hyperlipidemia, gallbladder stone, and ten kinds of anti-RA drugs (DMARD, TNF-blockers, corticosteroids).

^a^: Comparison group: patients without RA and matched with propensity score.

***p<0.001

As shown in Table 4, there was no significant dose–response effect seen with the use of glucocorticoids on reducing the risk of acute pancreatitis.

**Table 4:** Unadjusted HR and adjusted HR for risk of acute pancreatitis in RA patients with various PED dosages of corticosteroids.

PED^a^ (mg)	HR^b^ (95%CI^c^)	Adjusted HR (95%CI)
0 (reference)	1	1
0.01~5	0.62* (0.42~0.93)	0.57** (0.37~0.88)
5.01~10	0.68** (0.49~0.92)	0.71* (0.52~0.97)
10.01~15	0.58*** (0.42~0.80)	0.59*** (0.42~0.82)
≧15.01	0.73 (0.53~1.01)	0.71* (0.51~0.99)

Adjusted for age, gender, alcoholism, viral hepatitis, hyperlipidemia, gallbladder stone, and ten kinds of anti-RA drugs (DMARD, TNF-blockers, corticosteroids).

^a^PED: prednisolone equivalent dose

^b^HR: hazard ratio

^c^CI: confidential interval

*p<0.05

**p<0.01

***p<0.001

**Fig 2:**
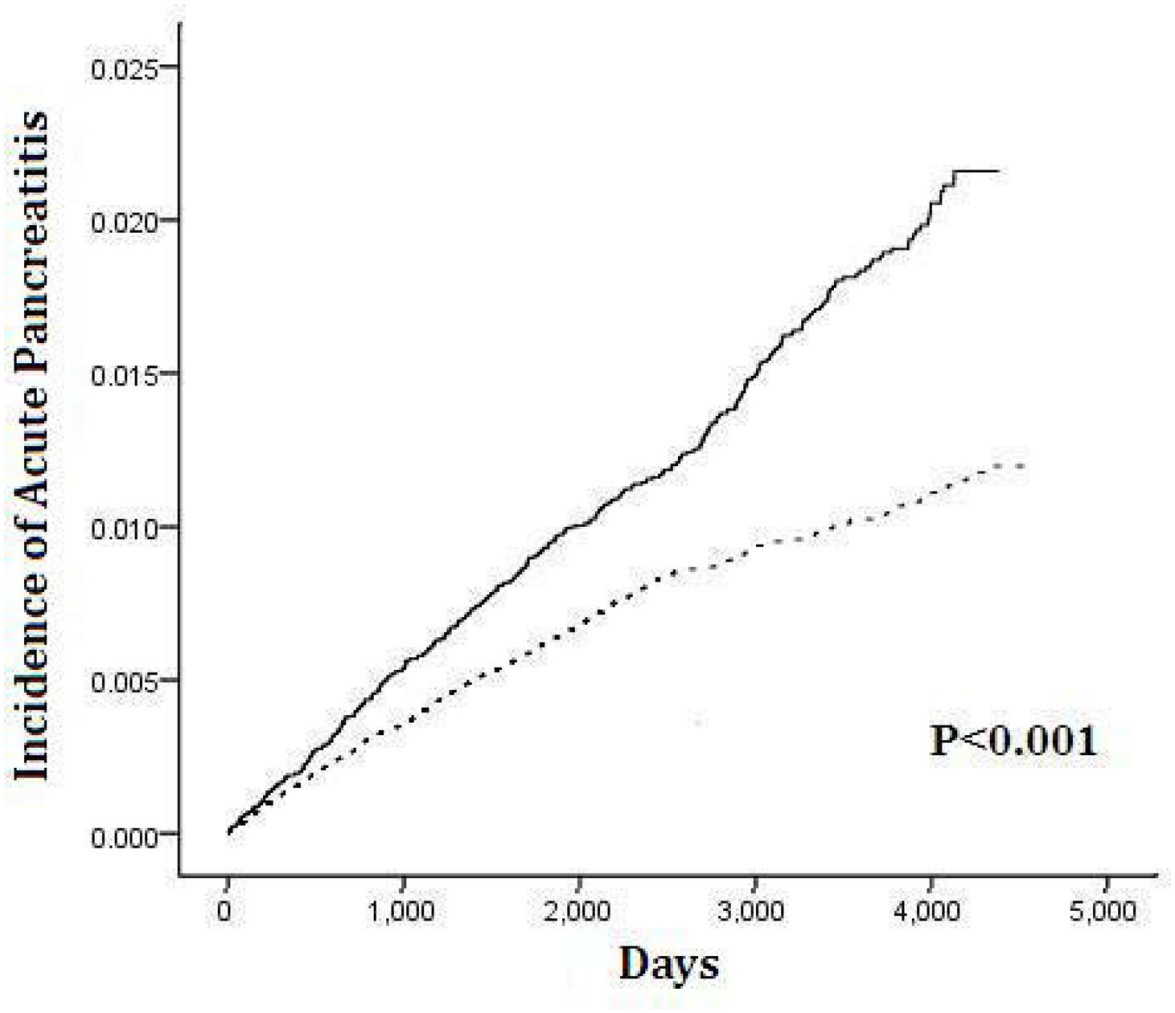
Kaplan-Meier curve of cumulative incidence of acute pancreatitis of RA patients and comparison groups. Footnotes: RA group,——; Comparison group,------; *P* < 0.001 by log-rank test.

The cumulative incidence of acute pancreatitis was higher in the RA group than in the non-RA group during the entire follow-up period, and the difference was significant based on the log-rank test (Fig 2).

## Discussion

To the best of our knowledge, this is the first population-based cohort study that simultaneously determined the risk of developing acute pancreatitis among RA patients and the effects associated with anti-RA drugs. In the present study, we demonstrated that patients with RA are at higher risk for acute pancreatitis. This study also revealed that oral glucocorticoids can reduce the risk, a finding that has not been previously reported in population-based cohort studies.

There are few studies that describe the risk of acute pancreatitis in patients with RA. In one autopsy study, the authors reported 6 cases with acute and 15 cases with chronic pancreatitis among 60 RA patients [27]. Furthermore, there have been several cases reports of fatal acute pancreatitis in RA patients [23–25]. The incidence of acute pancreatitis in RA patients found in our study was 185.7 per 100,000 person-years and an excess risk of 1.62 compared with non-RA patients. These studies suggest that patients with RA have an increased risk of acute pancreatitis, although Michaud et al [21] found that there is no evidence that the rate of pancreatitis is increased in RA, with or without treatment considerations. In addition, our observation shows that the excess hazard of acute pancreatitis continued to increase over time. This finding may support a causal association between RA and subsequent acute pancreatitis.

Among the RA patients identified from the database used in our study, 3,566 were inpatients and 26,189 were outpatients. For patients in the RA group who developed acute pancreatitis, 26 (0.1%) were outpatients and 371 (10.4%) were inpatients. This result suggested that there was a higher incidence of acute pancreatitis among patients with severe RA (those requiring hospitalization) compared with outpatient cases of RA.

Epidemiological studies have explored whether acute pancreatitis was associated with other comorbidities, particularly alcohol consumption, gallbladder stones, and viral hepatitis [4–6]. Some case studies have reported that interferon therapy in patients with chronic hepatitis C infection can induce acute pancreatitis [28–29]. In this study, patients with chronic hepatitis C infection received interferon therapy; however, no patient developed acute pancreatitis during the follow-up period (data not shown). There is no plausible biological explanation for the elevated risk of acute pancreatitis in RA patients with hepatitis C infection. However, RA remains as an independent factor for developing acute pancreatitis after adjusting for these potential risk factors.

The pathogenesis of acute pancreatitis in RA remains unknown. It is no doubt that an autoimmune mechanism may play an important role in its development. Bely et al [22] described autopsy results of a population of 161 inpatients with RA; they found 28 patients with pancreatitis and vasculitis characterized both histologically and immunohistochemically. Vasculitis of the pancreatic arterioles can lead to local ischaemia and to regressive changes in the pancreas. Vasculogenic, multifocal pancreatitis, involved the vessels and is followed by reactive inflammation. Many cases reports have also revealed amyloidosis in RA patients with fatal acute pancreatitis [23–25]. Authors draw attention to the frequent occurrence of pancreatitis in patients suffering from rheumatoid arthritis and the difficulties of a clinical diagnosis.

Autoimmune pancreatitis (AIP) is an increasingly recognized inflammatory condition of the pancreas characterized by increased levels of immunoglobulin (Ig) G4, the presence of autoantibodies, an association with other autoimmune diseases (Sjögren’s syndrome, rheumatoid arthritis, primary sclerosing cholangitis and inflammatory bowel disease) and a rapid response to corticosteroid therapy. AIP is believed to occur as a result of repeated overt or silent episodes of acute pancreatitis [30]. Three series have reported the prevalence of AIP as between 5 and 6% of all patients with chronic pancreatitis [31]. To date, this is the first study to report the magnitude of increased risk of acute pancreatitis in patients with RA and with the use of a retrospective cohort study design. Although in this study patients developed their first episode of acute pancreatitis after the diagnosis of RA, whether the condition will become chronic pancreatitis with repeated episodes requires additional study.

Some Canadian observational studies have demonstrated that corticosteroid use among patients with RA is between 30% and 40% [32–33]. In one Canadian study, it was found that the percentages of patients who used corticosteroids at any time were 74.4% and 58.5%, in an RA group and a control group, respectively [34]. The difference of exposure to corticosteroids in the two studied groups was similar to our study (89% vs. 76.5%). Another study estimated that corticosteroid use among patients with RA treated with biologics was 90% and for DMARDs, 75% [35]. The rate of corticosteroid use in this study is similar with that of our present research.

Our cohort design focused on evaluating whether taking anti-RA drugs could lower the risk of acute pancreatitis among patients with RA. Thus, our data analysis compared RA patients who had used and those who had not used a specific anti-RA drug. In the present study, RA patients taking oral glucocorticoids rather than DMARDs and TNF-blockers were found to have a reduction in the risk of acute pancreatitis. Limited studies have observed an association between anti-RA drugs and risk of acute pancreatitis.

This study has several strengths. It was based on a population-based study with a large sample size and an increased statistical testing significance. The large sample size increased the validity of identifying rare diseases (such as acute pancreatitis) for this study. The reduction of risk related to anti-RA drugs was also observed. Furthermore, the study group and comparison group were matched with a propensity score; this method can avoid selection bias of RA patients with more comorbidities than in the non-RA group.

Nevertheless, several limitations of the present study should also be addressed. First, a number of suspected risk factors of acute pancreatitis, such as cigarette smoking, were not available. This was due to the inherent limitation of the insurance data set. Second, the claims data of NHI do not provide data of laboratory tests. The disease activity and severity data of RA were also unavailable. This indicates a future study direction of whether the risk of acute pancreatitis is related to disease activity and severity level in RA. Finally, both the RA and acute pancreatitis diagnoses relied on administrative claims data reported by physicians and hospitals. These data may be less accurate than diagnoses made according to standardized criteria. However, we could not completely exclude the possibility of misdiagnosis. It is one of the major limitations in this type of study with the NIH database. However, the findings are sufficiently robust to prove that the RA can increase the risk of subsequent acute pancreatitis.

In conclusion, patients with RA are at an elevated risk of acute pancreatitis. Use of corticosteroids can reduce this risk. It should be the responsibility of the rheumatologist to take these risks and the risk of additional conditions into account when treating RA patients.

## References

[1] Fagenholz PJ, Castillo CF, Harris NS, Pelletier AJ, Camargo CA Jr. Increasing United States hospital admissions for acute pancreatitis, 1988–2003. Ann Epidemiol. 2007; 17:491–7. 17448682 10.1016/j.annepidem.2007.02.002

[2] Lowenfels AB, Maisonneuve P, Sullivan T. The changing character of acute pancreatitis: epidemiology, etiology, and prognosis. Curr Gastroenterol Rep. 2009; 11:97–103. 19281696 10.1007/s11894-009-0016-4

[3] Fu CY, Yeh CN, Hsu JT, Jan YY, Hwang TL. Timing of mortality in severe acute pancreatitis: experience from 643 patients. World J Gastroenterol. 2007; 13:1966–9. 17461498 10.3748/wjg.v13.i13.1966 PMC4146974

[4] Chang MC, Su CH, Sun MS, Huang SC, Chiu CT, Chen MC, et al Etiology of acute pancreatitis—a multicenter study in Taiwan. Hepatogastroenterology. 2003; 50:1655–7. 14571809

[5] Chen CH, Dai CY, Hou NJ, Chen SC, Chuang WL, Yu ML. Etiology, severity and recurrence of acute pancreatitis in southern Taiwan. J Formos Med Assoc. 2006; 105:550–5. 16877234 10.1016/S0929-6646(09)60149-2

[6] Jain P, Nijhawan S, Rai RR, Nepalia S, Mathur A. Acute pancreatitis in acute viral hepatitis. World J Gastroenterol. 2007; 13:5741–4. 17963301 10.3748/wjg.v13.i43.5741 PMC4171261

[7] Girman CJ, Kou TD, Cai B, Alexander CM, O'Neill EA, Williams-Herman DE, et al Patients with type 2 diabetes mellitus have higher risk for acute pancreatitis compared with those without diabetes. Diabetes Obes Metab. 2010; 12:766–71. 10.1111/j.1463-1326.2010.01231.x 20649628

[8] Nahon UK, Levy P, O’Toole D, Belmatoug N, Vullierme MP, Couvelard A, et al Is idiopathic chronic pancreatitis an autoimmune disease? Clin Gastroenterol Hepatol. 2005; 3:903–9. 16234029 10.1016/s1542-3565(05)00540-9

[9] Okazaki K, Uchida K, Ohana M, Nakase H, Uose S, Inai M, et al Autoimmune-related pancreatitis is associated with autoantibodies and a Th1/Th2-type cellular immune response. Gastroenterology. 2000; 118:573–81. 10702209 10.1016/s0016-5085(00)70264-2

[10] Aparisi L, Farre A, Gomez-Cambronero L, Martinez J, De Las Heras G, Corts J, et al Antibodies to carbonic anhydrase and IgG4 levels in idiopathic chronic pancreatitis: relevance for diagnosis of autoimmune pancreatitis. Gut. 2005; 54:703–9. 15831920 10.1136/gut.2004.047142 PMC1774474

[11] Uchida K, Okazaki K, Nishi T, Uose S, Nakase H, Ohana M, et al Experimental immune-mediated pancreatitis in neonatally thymectomized mice immunized with carbonic anhydrase II and lactoferrin. Lab Invest. 2002; 82:411–24. 11950899 10.1038/labinvest.3780435

[12] Davidson TS, Longnecker DS, Hickey WF. An experimental model of autoimmune pancreatitis in the rat. Am J Pathol. 2005; 166:729–36. 15743785 10.1016/S0002-9440(10)62294-8 PMC1602363

[13] Ghazale A, Chari ST. Optimising corticosteroid treatment for autoimmune pancreatitis. Gut. 2007; 56:1650–2. 17998320 10.1136/gut.2007.129833 PMC2095689

[14] Kolk A, Horneff G, Wilgenbus KK, Wahn V, Gerharz CD. Acute lethal necrotizing pancreatitis in childhood SLE-possible toxicity of immunosuppressive therapy. Clin Exp Rheumatol. 1995; 13:399–403 7554572

[15] Eisemann AD, Becker NJ, Miner PB Jr, Fleming J. Pancreatitis and gold treatment of rheumatoid arthritis. Ann Int Med. 1989; 111(10):860–1. 10.7326/0003-4819-111-10-860_2 2573305

[16] Hamed I, Lindeman RD, Czerwinski AW. Acute pancreatitis following corticosteroid and azathioprine therapy. J Med Sci. 1978; 276:211–9. 10.1097/00000441-197809000-00009 736057

[17] Paloyan D, Levin B, Simonowitz D. Azathioprine-associated acute pancreatitis. Am J Dig Dis. 1977; 22:839–40. 900102 10.1007/BF01694518

[18] Rodrı´guez-Rodrı´guez L, Lamas JR, Varade´ J, Tornero-Esteban P, Abasolo L, de la Concha EG, et al Combined influence of genetic and environmental factors in age of rheumatoid arthritis onset. Rheumatol Int. 2012; 32:3097–102. 21922340 10.1007/s00296-011-2090-9

[19] Firestein GS. Evolving concepts of rheumatoid arthritis. Nature. 2003; 423:356–61. 12748655 10.1038/nature01661

[20] Lai CH, Lai MS, Lai KL, Chen HH, Chiu YM. Nationwide population-based epidemiologic study of rheumatoid arthritis in Taiwan. Clin Exp Rheumatol. 2012; 30:358–63. 22513120

[21] Michaud K, Wolfe F. Comorbidities in rheumatoid arthritis. Best Pract Res Clin Rheumatol. 2007; 21:885–906. 17870034 10.1016/j.berh.2007.06.002

[22] Bély M, Apáthy A. Recurrent pancreatic arteritis and vasculogenic relapsing pancreatitis in rheumatoid arthritis—a retrospective clinicopathologic and immunohistochemical study of 161 autopsy patients. Pathol Oncol Res. 2008; 14:473–80. 10.1007/s12253-008-9026-z 18975138

[23] Oishi K, Wada J, Nagake Y, Hida K, Hashimoto H, Hayakawa N, et al Fatal pancreatitis associated with systemic amyloidosis in a rheumatoid arthritis patient. J Med. 2000; 31:303–10. 11508323

[24] Kuroda T, Sato H, Hasegawa H, Wada Y, Murakami S, Saeki T, et al Fatal acute pancreatitis associated with reactive AA amyloidosis in rheumatoid arthritis with end-stage renal disease: a report of three cases. Intern Med. 2011; 50:739–44. 21467708 10.2169/internalmedicine.50.4876

[25] Ichikawa T, Nakao K, Hamasaki K, Ohkubo K, Toriyama K, Eguchi K. An autopsy case of acute pancreatitis with a high serum IgG4 complicated by amyloidosis and rheumatoid arthritis. World J Gastroenterol. 2005; 11:2032–4. 15801001 10.3748/wjg.v11.i13.2032 PMC4305732

[26] Lanehart RE, de Gil PR, Kim ES. Propensity score analysis and assessment of propensity score approaches using SAS procedures. SAS Global Forum: Statistics and Data Analysis 2012; Paper 314–2012.

[27] Bély M, Apáthy A, Pintér T, Ratkó I.Pancreatitis in rheumatoid arthritis found in autopsy material. Morphol Igazsagugyi Orv Sz. 1989; 29:213–21. 2677691

[28] Eland IA, Rasch MC, Sturkenboom MJ, Bekkering FC, Brouwer JT, Delwaide J, et al Acute pancreatitis attributed to the use of interferon alfa-2b. Gastroenterology. 2000; 119:230–3. 10889173 10.1053/gast.2000.8528

[29] Chaudhari S, Park J, Anand BS, Pimstone NR, Dieterich DT, Batash S, et al Acute pancreatitis associated with interferon and ribavirin therapy in patients with chronic hepatitis C. Dig Dis Sci. 2004; 49:1000–6. 15309891 10.1023/b:ddas.0000034562.17003.50

[30] Vonlaufen A, Wilson JS, Apte MV. Molecular mechanisms of pancreatitis: current opinion. J Gastroenterol Hepatol. 2008; 23:1339–48. 10.1111/j.1440-1746.2008.05520.x 18853993

[31] Kim KP, Kim MH, Song MH, Lee SS, Seo DW, Lee SK. Autoimmune chronic pancreatitis. Am J Gastroenterol. 2004; 99:1605–16. 15307882 10.1111/j.1572-0241.2004.30336.x

[32] McKeown E, Bykerk VP, De LF. Quality assurance study of the use of preventative therapies in glucocorticoid-induced osteoporosis in early inflammatory arthritis: results from the CATCH cohort. Rheumatology (Oxford). 2012; 51:1662–9. 22539481 10.1093/rheumatology/kes079

[33] vina-Zubieta JA, Abrahamowicz M, De Vera MA. Immediate and past cumulative effects of oral glucocorticoids on the risk of acute myocardial infarction in rheumatoid arthritis: a population-based study. Rheumatology (Oxford). 2013; 52:68–75. 23192907 10.1093/rheumatology/kes353

[34] Dixon WG, Abrahamowicz M, Beauchamp ME. Immediate and delayed impact of oral glucocorticoid therapy on risk of serious infection in older patients with rheumatoid arthritis: a nested case-control analysis. Ann Rheum Dis 2012; 71:1128–33. 10.1136/annrheumdis-2011-200702 22241902 PMC3375584

[35] Wu CY, Chen DY, Shen JL.The risk of cancer in patients with rheumatoid arthritis taking tumor necrosis factor antagonists: a nationwide cohort study. Arthritis Res Ther. 2014; 16:449 10.1186/s13075-014-0449-5 25267341 PMC4201718

